# Interactive online tool to identify genes associated with longevity in a panel of long-lived genetic mutants in *C. elegans*

**DOI:** 10.17912/micropub.biology.002153

**Published:** 2026-07-01

**Authors:** John N. Hutchinson, Jiaxi Guan, Grant F. Booth, Jeremy M Van Raamsdonk

**Affiliations:** 1 Diamond Age Data Science, Somerville, Massachusetts, USA; 2 Harvard Chan Bioinformatics Core, Department of Biostatistics, Harvard T.H. Chan School of Public Health, Boston, Massachusetts, USA; 3 Department of Neurology and Neurosurgery, McGill University, Montreal, Quebec, Canada; 4 Metabolic Disorders and Complications Program, Research Institute of the McGill University Health Centre, Montreal, Quebec, Canada; 5 Brain Repair and Integrative Neuroscience Program, Research Institute of the McGill University Health Centre, Montreal, Quebec, Canada; 6 Division of Experimental Medicine, Department of Medicine, McGill University, Montreal, Quebec, Canada

## Abstract

We recently used RNA sequencing to identify differentially expressed genes in a panel of nine long-lived mutants in *C. elegans* representing multiple different pathways of lifespan extension. In order make this data accessible to the research community, we have developed an online tool that allows users to examine the expression of a gene of interest in individual long-lived mutants or across all nine long-lived mutants. This tool will enable users to gain insight into how their genes of interest are modulated in long-lived genetic mutants and the extent to which expression of that gene is correlated with long life.

**Figure 1. Sample Outputs from Gene Lookup Shiny App.** This figure is showing the output for the expression of the gene *cdr-2* in *clk-1, isp-1* and *nuo-6* mutants. (A) The “TSM Plots” tab will display a graph comparing the expression levels of the selected gene(s) in the selected strain(s) to the expression in wild-type worms. All individual values are shown as well as the average. (B) The “Statistics” tab will show whether there are significant differences in the expression of the selected gene(s) from wild-type worms including the fold change (log2) and adjusted p-value (padj). It also displays the mean and standard deviation for each strain. (C) The “TPMs” tab will display the transcripts per million for each individual sample for the gene(s) and strain(s) selected.

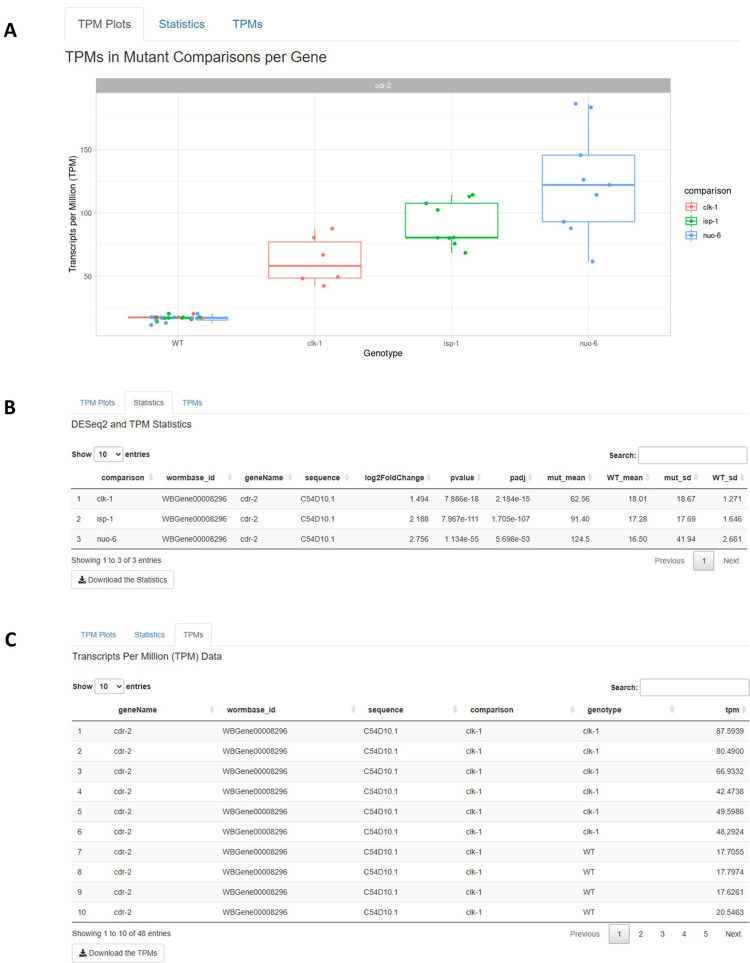



## Description

Aging can be described as the progressive loss of function at both a molecular and cellular level driven by the accumulation of molecular damage and altered molecular signaling leading to an overall decline in an organism’s ability to repair and maintain itself (Li et al., 2024). Aging is the greatest risk factor for the development of a multitude of chronic diseases including age-onset neurodegenerative disorders. As a result, the geroscience hypothesis proposes that by gaining insight into the aging process, this knowledge can be used to mitigate multiple chronic diseases simultaneously thereby allowing individuals to live healthier, longer lives. One of the main goals of geroscience is to advance our understanding of the biology of aging to identify novel molecular targets to modulate the aging process. 

One approach that has been used to elucidate the molecular mechanism contributing to aging is RNA sequencing (RNA-seq). RNA-seq provides a genome-wide, unbiased method to characterize global gene expression patterns associated with aging and longevity (Bairakdar et al., 2023; Mosley et al., 2025). Rather than focusing on individual candidate genes, RNA-seq enables the identification of coordinated transcriptional changes, pathway-level alterations, and gene expression programs that reflect broader biological states (Marguerat and Bahler, 2010; Wang et al., 2009). In this way, different long-lived mutants or lifespan-extending interventions can be defined by characteristic transcriptomic signatures or expression profiles (Tyshkovskiy et al., 2023). Such comparative transcriptomic analysis provides a data-driven framework to better understand the molecular basis of aging and to generate new mechanistic hypotheses.

To gain insight into shared and unique molecular mechanisms contributing to lifespan extension, we used RNA-seq to examine gene expression in a panel of nine long-lived mutants that represent multiple biological pathways known to regulate lifespan in *C. elegans* . Reduced insulin/IGF-1 signaling was examined using the *daf-2(e1370)* mutant, one of the earliest and most well-characterized longevity models (Kenyon et al., 1993). Dietary restriction was modeled using *eat-2(ad1116)* mutants, which exhibit extended lifespan due to reduced food intake (Lakowski and Hekimi, 1998). Decreased protein translation was represented by *ife-2(ok306)* mutants (Syntichaki et al., 2007), while altered chemosensory signaling was examined using *osm-5(p813)* mutants (Apfeld and Kenyon, 1999). Germline ablation–associated longevity was modeled using *glp-1(e2141)* mutants (Hsin and Kenyon, 1999). To assess mild impairment of mitochondrial function, we included *clk-1(qm30)* , *isp-1(qm150)* , and *nuo-6(qm200)* mutants (Feng et al., 2001; Lakowski and Hekimi, 1996; Yang and Hekimi, 2010). Finally, we used *sod-2(ok1030)* mutants, which have increased levels of mitochondrial superoxide (Van Raamsdonk and Hekimi, 2009). Together, these models represent multiple independent genetic perturbations that lead to lifespan extension, enabling comparative analysis of different longevity strategies.

In comparing differentially expressed genes across the panel of nine long-lived mutants, we identified a number of genes that are upregulated or downregulated in at least six long-lived mutants but relatively few in seven or more (Rudich et al., 2025). The commonly upregulated genes were enriched for genes involved in immunity, defense and metabolism, while downregulated genes were enriched for genes involved in translation and gene expression. Interestingly, we found that the nine long-lived mutants clustered into three different longevity groups according to their gene expression. Group 1 longevity mutants contained *daf-2, glp-1, clk-1, isp-1, nuo-6* and *sod-2* ; group 2 contained *eat-2* and *osm-5* ; and group 3 contained *ife-2.* Group 1 and group 2 longevity mutants exhibited modulation of specific genetic pathways in opposite directions (Rudich et al., 2025). For example, target genes of the DAF-16-mediated stress response pathway and mitochondrial unfolded protein response are significantly upregulated in group 1 longevity mutants but downregulated or unchanged in group 2 longevity mutants. This suggests that longevity can be achieved through multiple distinct molecular strategies.

To make our gene expression data accessible to the research community and facilitate the identification of genes associated with longevity, we created an interactive web-based Shiny application that enables exploration of RNA-seq gene expression across multiple long-lived *C. elegans* mutants relative to wild-type controls. The application can be found here: https://vanraamsdonk.shinyapps.io/mutant_comparison_viewer/ . Users can select one or more mutants for comparison, including *clk-1, daf-2, eat-2, glp-1, ife-2, isp-1, nuo-6, osm-5,* and *sod-2* . The application accepts one or multiple genes as input and supports common gene names (e.g., aap-1), WormBase gene IDs (e.g., WBGene00000001), and sequence names (e.g., Y110A7A.10). This interface allows rapid cross-model comparison of gene expression across distinct longevity pathways without requiring programming expertise. All datasets shown in the app were processed using a standardized RNA-seq pipeline (bcbio-nextgen: https://zenodo.org/records/5781867 ). Differential expression was computed with DESeq2 (with shrinkage) (Love et al., 2014), and transcripts per million (TPM) values were derived from Salmon-based quantification.

To use the application, users first select one or more mutant strains under the “Select Comparisons” panel (For screenshots on how to use the application see **Extended** **Figure 1** ). Genes of interest are then entered into the input field using gene names, WormBase gene IDs, or sequence names. After submitting the query, the application automatically generates expression plots and corresponding statistical summaries for the selected strains relative to wild-type controls.

The application displays gene expression results in three main formats ( **Figure 1** ). The “TSM Plots” tab shows expression levels for each selected mutant compared to wild-type controls, including individual sample values and the average for each strain. This allows users to see the direction of change and the variation within each group. The “Statistics” tab provides quantitative results, including log2 fold change and adjusted p-value (padj), which indicate the magnitude and statistical significance of the difference. The “TPMs” tab lists the TPM values for each individual sample, allowing users to view the underlying expression levels. Together, these outputs help users evaluate whether a gene is consistently increased or decreased across different longevity mutants.

In addition to visualization and summary statistics, the application provides access to the full underlying datasets ( **Extended** **Figure 2** ). Under the “Datasets” tab, users can view sample metadata, differential expression statistics generated by DESeq2, and TPM values for all genes. The DESeq2 table includes log2 fold change, p-values, adjusted p-values, and expression means for each strain compared to wild type. These tables can be downloaded, allowing users to perform further analysis outside of the web interface. Access to the full datasets makes it possible to further explore similarities and differences in gene regulation across distinct longevity pathways.

In conclusion, this tool provides a simple way to compare gene expression across multiple long-lived mutants within a single framework. By examining the same gene across different longevity pathways, researchers can identify shared changes as well as pathway-specific patterns of regulation. This approach may help guide further experiments aimed at understanding how distinct genetic interventions influence aging at the molecular level.

## Methods

Strains were maintained on NGM plates with OP50 bacteria at 20°C. Worms were collected for RNA sequencing at the pre-fertile young adult stage. A minimum of six biological replicates per strain were used. Each biological replicate consisted of a separate plate of worms generated by a limited lay on a different day than the other replicates. RNA was isolated using TRIZOL as described previously (Machiela et al., 2016). Sequencing libraries were generated with the Kapa Biosystems stranded mRNA-Seq kit for the Illumina platform. Libraries were sequenced using 1×75 bp sequencing on the Illumina NextSeq 500 platform to a depth of ∼30M reads per sample (Dues et al., 2017). All samples were processed using an RNA-seq pipeline implemented in the bcbio-nextgen project ( https://bcbio-nextgen.readthedocs.org/en/latest/ ). Raw reads were examined for quality issues using FastQC ( http://www.bioinformatics.babraham.ac.uk/projects/fastqc/ ) to ensure library generation and sequencing are suitable for further analysis. Adapter sequences, other contaminant sequences such as polyA tails and low quality sequences with PHRED quality scores less than five were trimmed from reads using atropos ( https://github.com/jdidion/atropos ; 10.5281/zenodo.596588). Trimmed reads were aligned to the *C. elegans* genome, augmented with transcript information from Ensembl using STAR (Dobin et al., 2013). Alignments were checked for evenness of coverage, rRNA content, genomic context of alignments (for example, alignments in known transcripts and introns), complexity and other quality checks using a combination of FastQC, Qualimap (Garcia-Alcalde et al., 2012), MultiQC ( https://github.com/ewels/MultiQC ) and custom tools. Counts of reads aligning to known genes were generated by featureCounts (Liao et al., 2014) and used for additional quality checks. In parallel, Transcripts Per Million (TPM) measurements per isoform were generated by quasi-alignment using Salmon (Patro et al., 2017). Differential expression at the gene level was called with DESeq2 (Love et al., 2014) using shrinkage. In brief, the largest fold changes not due to low counts were used inform a prior distribution and shrink fold changes that lack statistical information. Counts per gene were estimated from the Salmon quasi-alignments by tximport (Soneson et al., 2015) as quantitating at the isoform level has been shown to produce more accurate results at the gene level.

## Reagents

The following worm strains were used:

**Table:** 

**Original Strain Name**	**Genotype**	**Source**
N2	wild-type	CGC
KX15	*ife-2 (ok306)*	CGC
MQ130	*clk-1(qm30)*	Hekimi lab
MQ1451	*sod-2(ok1030)*	Hekimi lab
MQ631	*eat-2(ad1116)*	CGC
PR813	*osm-5(p813)*	CGC
MQ1333	*nuo-6(qm200)*	Hekimi lab
MQ887	*isp-1(qm150)*	Hekimi lab
CB1370	*daf-2(e1370)*	CGC
CB4037	*glp-1(e2141)*	CGC

The application can be accessed here: https://vanraamsdonk.shinyapps.io/mutant_comparison_viewer/

## Data Availability

Description: Software release github zipfile.. Resource Type: Software. DOI: https://doi.org/10.22002/8vmke-tn046 Description: Extended Figure 1, 2. Resource Type: Image. DOI: https://doi.org/10.22002/fq62m-ypz91
